# Supplementary material for: Effects of an 8-week yoga program on sustained attention and discrimination function in children with attention deficit hyperactivity disorder
Source: PeerJ. 2017 Jan 12;5:e2883. doi: 10.7717/peerj.2883 (PMC5237364; doi:10.7717/peerj.2883)
Supplement: Supplemental Information 2 [file peerj-05-2883-s002.docx]

**The Study Protocol**

**Research Title: Effects of an 8-Week Yoga Program on Sustained Attention and Discrimination Function in Children with Attention Deficit Hyperactivity Disorder**

**General information**

**Principal Investigator: ChienChih Chou**

Address: No.101, Sec. 2, Zhongcheng Rd., Shilin Dist., Taipei City

Telephone: +886- 2-2871-8288

Fax: +886- 2-2875-1143

E-mail address: ccc4453@gmail.com

Main responsibilities/Key roles: He was the primary individual responsible for the preparation, conduct, and administration of the research grant for the project of the children yoga exercise program on the effects of executive function, cooperative agreement, training or contract in compliance with applicable laws and regulations and institutional policy governing the conduct of sponsored research.

**Co-Investigators: Chung-Ju Huang**

Address: No.101, Sec. 2, Zhongcheng Rd., Shilin Dist., Taipei City

Telephone: +886- 2-2871-8288

Fax: +886- 2-2875-1143

e-mail address: crhwang_tpec@yahoo.com.tw

Main responsibilities/Key roles: Co-Investigator was a key personnel who had responsibilities similar to that of a Principal Investigator on the project of the yoga exercise program on the effects of executive function. While the Principal Investigator had ultimate responsibility for the conduct of a research project, the Co-Investigator was also obligated to ensure the project was conducted in compliance with applicable laws and regulations and institutional policy governing the conduct of sponsored research.

***Rationale & background information***

Physical activity intervention has been found to derive positive changes in behavioral structures and cognitive function among children with ADHD, which are reflected in reduced impulsivity, anxiety, and improved attention (Chang, Hung, Huang, Hatfield, & Hung, 2014; Huang et al., 2014; Smith et al., 2013; Verret, Guay, Berthiaume, Gardiner, & Béliveau, 2012). In particular, the mechanisms of physical activity effects on ADHD children’s cognitive function may be due to brain structure changes and enhanced neurotransmitters (Lustig, Shah, Seidler, Reuter-Lorenz; 2009; Tang, Chu, Hui, Helmeste, & Law, 2008). Like physical activity intervention, yoga has been found to have beneficial impacts on neurological and physiological activity and behavior in a range of populations. The reported benefits of yoga include increased slow-frequency brain wave activity (Arambula, Peper, Kawakami, & Gibney, 2001); favorable profiles on heart rate variability, depression, perceived stress, and superior aerobic fitness (Satin, Linden, & Millman, 2014); significant decrement of cortisol and increment in brain-derived neurotropic factor (BDNF), serotonin, and dopamine (Pal, Singh, Chatterjee, & Saha, 2014); decreased teacher ratings of maladaptive behavior (Koenig, Buckley-Reen, & Garg, 2012). In essence, the practice of yoga exercise elicits reduced activation of the sympathetic nervous system and increased activation of the parasympathetic nervous system resulting in a sense of equilibrium into the body and mind, and increased concentration (Brosnan, 1982). Given that abnormal attention and over-impulsivity characteristics have been considered as major symptoms of ADHD, these previous studies provide compelling empirical evidence for using yoga exercise in ADHD treatment.

Generally, the symptoms of ADHD are associated with the inability to inhibit behavioral impulsivity, sustain attention, and correctly differentiate and react to rapidly changing and continuous external stimuli. Any changes in sustained attention and discrimination function observed after yoga exercise could be used to identify the effects of yoga on improved ADHD symptoms.

***References (of literature cited in preceding sections)***

Pal, R., Singh, S. N., Chatterjee, A., & Saha, M. (2014). Age-related changes in cardiovascular system, autonomic functions, and levels of BDNF of healthy active males: Role of yogic practice. Age, 36(4):9683. doi: 10.1007/s11357-014-9683-7

Koenig, K. P., Buckley-Reen, A., & Garg, S. (2012). Efficacy of the get ready to learn yoga program among children with autism spectrum disorders: A pretest-posttest control group design. American Journal of Occupational Therapy, 66, 538-546. doi:10.5014/ajot.2012.004390

Brosnan. B. (1982). Yoga for handicapped people. London: Souvenir Press.

Chang, Y. K., Hung, C. L., Huang, C. J., Hatfield, B. D., & Hung, T. M. (2014). Effects of an aquatic exercise program on inhibitory control in children with ADHD: A preliminary study. Archives of Clinical Neuropsychology, 29, 217-223. doi:10.1093/arclin/acu003

Hung, C. L., Chang, Y. K., Chan, Y. S., Shih, C., Huang, C., & Hung, T. (2013). Motor ability and inhibitory processes in children with ADHD: A neuroelectric study. Journal of Sport & Exercise Psychology, 35, 322-328.

Verret, C., Guay, M., Berthiaume, C., Gardiner, P., & Béliveau, L. (2012). A physical activity program improves behavior and cognitive functions in children with ADHD: An exploratory study. Journal of Attention Disorders, 16, 71-80. doi: 511 10.1177/1087054710379735

Lustig, C., Shah, P., Seidler, R., & Reuter-Lorenz, P. A. (2009). Aging, training, and the brain: A review and future directions. Neuropsychology Review, 19, 504-522. doi: 10.1007/s11065-009-9119-9

Tang, S. W., Chu, E., Hui, T., Helmeste, D., & Law, D. (2008). Influence of exercise on serum brain-derived neurotrophic factor concentrations in healthy human subjects. Neuroscience Letters, 431, 62-65. doi: 10.1016/j.neulet.2007.11.019.

Arambula, P., Peper, E., Kawakami, M., & Gibney, K. H. (2001). The physiological correlates of Kundalini yoga meditation: A study of a yoga master. Applied Psychophysiology and Biofeedback, 26, 147-153.

Satin, J. R., Linden, W., & Millman, R. D. (2014). Yoga and psychophysiological determinants of cardiovascular health: Comparing yoga practitioners, runners, and sedentary individuals. Annals of Behavioral Medicine, 47, 231-241. doi: 10.1007/s12160-013-9542-2

***Study goals and objectives***

The goal of this research was to investigated whether a yoga exercise intervention influenced the sustained attention and discrimination function in children with ADHD.

***Study Design***

It was hypothesized that yoga exercise could benefit the sustained attention and discrimination function of children with ADHD by using the Visual Pursuit Test and the Determination Test. The research to be conducted under this protocol falls under the intervention of yoga exercise activity on the sustained attention and discrimination function for the children with ADHD (executive function), which contrasts behaviors in simple sedentary environment with predictions of executive function theory. Examples of the experiments include stretching, warming-up, concentration, and balance, attention, breath, body awareness, balancing, flexibility, and relaxation exercises.

Yoga exercise activity was manipulated to identify factors that influence children with ADHD to improve sustained attention and discrimination function that are either more accuracy rate or decreasing reaction time (sec.). The manipulation of yoga activity followed the American Physical Therapy Association guidelines for working with children. During the entire session, each participant’s HR was recorded at one-min intervals by using a Polar HR monitor (Mode ZW 60 GT5; Cardiosprt, United Kingdom). The HR values during the periods of warming-up, main activity, and cooling-down were calculated respectively. During the main activity for the yoga exercise, the intensity of exercise was approximately 53% HRmax (220 - age). Briefly stated, for the experimental group, the yoga activity induced arousal which was then slightly reduced after the yoga exercise was ceased.

The protocol will be implemented in two setting: 1) executive function laboratory, or 2) in a dance studio. The lab-based experiments will be the more complex and longer experiments, while the experiments executed at dance studio will be an average temperature of 24°-26°C. All of the activities about the particular yoga exercise, including stretching, warming-up, concentration, and balance, attention, breath, body awareness, balancing, flexibility, and relaxation exercises will be provided to participants during they involve.

***Methodology***

The researchers provided participants about outline of and a rationale for the research design. The researchers also provided the details of the research method to be employed and any combinations of techniques or methods (e.g. Visual Pursuit Test, the Determination Test, and a physical fitness assessment). The researchers were clearly indicated that the issues associated with the use of the methods being advocated are fully understood. The participants were given to full explanation of tests and data collection procedures which sets out in detail:

1. ***Participants***

***Recruitment and inclusion / exclusion criteria:*** The inclusion criteria were as follows: children aged between 8 and 12 years old who had been diagnosed with ADHD by a psychiatric physician as well as by a school pediatrician according to the DSM-IV criteria and interviews with parents. The exclusion criteria were as follows: a) comorbid conditions such as conduct/oppositional defiant disorder, autism spectrum disorders, or serious affective disorders, b) a personal history of brain injury or neurological disorders, and c) currently taking sedatives or other mood altering medications other than the stimulants typically prescribed for ADHD. Moreover, all the various subtypes of ADHD (inattentive, hyperactivity/impulsivity, combined) were included so long as the participant met the other criteria, and regardless of whether he or she was receiving medication for it.

1. ***Measurement***

***Visual Pursuit Test***: The Visual Pursuit Test of the Vienna Test System (Schuhfried GmbH, Austria), a computerized psychological assessment tool well established in psychological diagnostics, was used in this study. It is designed as a line tracking test and used for the registration of concentrated targeted perception and selective attention in the visual area. Hence, the performance in this test requires the ability of selective and sustained attention. It consisted of 54 different items; in each item, an array of nine entwined dark lines leading to nine different endpoints was presented on a light background computer screen. The performance of the participant was scored automatically, considering the number of correct answers and mean RT for correct answers. The test duration for each participant was approximately 10 min per run.

***Determination Test***: The Determination Test has been used to assess the discrimination ability for reaction speed, attention deficits, and reactive stress tolerance in the presence of continuous but rapidly changing acoustic and optical stimuli (Shmygalev et al., 2011). In this study, the participant’s task was to react as quickly as possible to visual or acoustic stimuli by pressing the corresponding buttons on the response panel. There were five visual stimuli colored white, yellow, red, green and blue, which appeared in an upper and a lower row on the screen. This test contained 180 trials with 20 trials for each stimulus. The number of correct trials was utilized to calculate the accuracy rate and RT of each correct response was reported. The duration for the test was approximately 10 min for each participant.

***Physical Fitness*:** The participants were instructed to not engage in any intense physical activity or take any stimulant medication on the day before the evaluations. In addition to weight and height, the physical fitness of each participant was estimated, including flexibility, muscular endurance, power, and cardiovascular fitness. The fitness assessment includes measures of flexibility (sit and reach test), muscular endurance (sit-ups in 1 min), power (standing long jump), and cardiovascular fitness (a half-mile run in the fastest possible time).

1. ***Intervention and procedure***

***Yoga Exercise Intervention:*** The manipulation of yoga activity followed the American Physical Therapy Association guidelines for working with children (Galantino, Galbavy, & Quinn, 2008). Each lesson for yoga activity lasted for 40 min, twice a week for eight weeks, was led by a nationally certified yoga instructor, and was conducted in a dance studio with an average temperature of 24-26°C. The yoga activity session consisted of a 10-minute stretching and warming-up period followed by a 20-minute yoga activity, which included concentration and balance, improved attention, and breath and body awareness. Finally, each session ended with a 10-minute cooling-down period including balancing, flexibility, and relaxation exercises. During the entire session, each participant’s HR was recorded at one-min intervals by using a Polar HR monitor (Mode ZW 60 GT5; Cardiosprt, United Kingdom). The HR values during the periods of warming-up, main activity, and cooling-down were calculated respectively.

***Procedure***: The participants were invited to come to the laboratory with their parents on two separate days. The children who were undergoing medical treatment were asked to refrain from medication for at least 24 h prior to the experiment. On the first visit, the participant’s parent(s) and the participant signed an informed consent form, provided a health history, and filled out a demographics questionnaire. Each eligible participant then entered the pre-test stage, which consisted of performing the Visual Pursuit Test and the Determination Test and a physical fitness assessment. The order of the Visual Pursuit Test and the Determination Test was counterbalanced. Participants in the yoga exercise group underwent an eight-week yoga exercise program that consisted of two 40-min sessions per week as an after school program. In contrast to the exercise group, the participants in the control group were simply instructed to maintain their normal life without participating in regular physical activity programs. Within one week of completing the yoga exercise program, all the participants were invited to visit the laboratory for the second time. Each participant was asked to perform the Visual Pursuit Test and the Determination Test again for the comparison of pre- and post-test scores.

***Safety Considerations***

The wellbeing and safety of participants in research, including children who participate in research, are the paramount considerations at all times. The protection of research participants takes precedence above all other consideration including the potential for our study to contribute to new knowledge in physical education.

***For the delivery of the manipulated yoga activity protocol:*** The researchers offered a pragmatic perspective on the need for a yoga instructor to have specialized teaching qualifications in yoga.

***Safety procedure:*** Since the yoga program was conducted by an experienced instructor, we do not expect any emotional interference to classroom management or other children in learning period. However, to err on the side of precaution, participant's parents had access to Dr. ChienChih Chou's cell phone number directly, which was active 24 hours a day. Moreover, any indications of severe psychological distress during the yoga activity will be explored and, if necessary, appropriate referrals for children's mental section were made in the Department of Student Counseling, University of Taipei.

***Data Management and Statistical Analysis***

***Project Information***

This Data Management Plan (DMP) for our study covers the data which were collected in elementary schools of Taipei city. The study projected to be conducted between May 13, 2013 and June 30, 2014. The study collected non-sensitive data from children with attention deficit hyperactivity disorder subjects ranging from 8 to 12 years of age. No other personal identifiers would be collected during the study apart from those identified above.

The data collected during this study were archived with the Graduate Institute of Sport Pedagogy at the University of Taipei. The data would be stored in a specific virtual archive in the Lab of Sport Pedagogy at the University of Taipei and is made publicly available through the Graduate Institute of Sport Pedagogy, Dr. Chou, Chien-Chih who is in charge for the data management. The Graduate Institute of Sport Pedagogy Data Archive is a well-established and trusted archive in the social science field.

***General Data Management Plan information***

This Data Management Plan was created and approved on April 24, 2014 for submission to the Graduate Institute of Sport Pedagogy as required by the Research Ethics Committee, National Taiwan University guidelines. The aim and purpose of this Data Management Plan is to detail and guarantee the preservation of the data collected during this study, as well as any results derived from the associated research. This Data Management Plan is intended for review by relevant Research Ethics Committee, National Taiwan University personnel, as well as the Lab of Sport Pedagogy staff affiliated directly with this study and the collection and preservation of the associated data and research. This is the first iteration of this Data Management Plan associated with this data.

***Policies***

There were no requirements stipulated by the funding or partner organizations regarding this data. Comprehensive institutional and research group guidelines specified by the University of Taipei were applied regarding the collection of this data. There are no additional requirements associated with the data being submitted.

***Legal Guidelines and Requirements***

This study was only collected non-sensitive data (e.g. Blood Test, Physiological Test, Invasive test, Invasive Treatment...et al.) from children with attention deficit hyperactivity disorder. No personal identifiers were recorded or retained by the researchers in any form. There were no copyright or licensing issues associated with the data being submitted.

***Data Standards and Collect***

The associated data types were collected using the computer version of Visual Pursuit Test and Determination Test and analyzed using SPSS data analytics tools. The researchers were not aware of any issues regarding the effects or limitations of these formats regarding the data being submitted.

***Security, Storage, Management and Back-Up of Data***

The Lab of Sport Pedagogy’s experience with, and commitment to, secure data archiving is well established and is in keeping with established the University of Taipei Information Security Policies. During the implementation of the tests, associated research data was physically stored on a password-protected secure server maintained by Graduate Institute of Sport Pedagogy, University of Taipei using standard SPSS file formats. No data were reside on portable or laptop devices, and no other external media/format(s) were used for data storage. Research data was backed up on a daily basis. The researchers are currently responsible for storage, maintenance and back-up of the data. The specific storage volume of the data being submitted was not more than 2 GB maximum. The long-term strategy for the maintenance and archiving of the data would be implemented when the data and associated research are migrated to the Institute of Sport Pedagogy, University of Taipei for archiving.

***Ethics***

The implementation of this Protocol, through the Research Review Committee, was guided in accordance to the values and protocols of the National Taiwan University.

***Quality Assurance (QA)***

The researchers are responsible for the management, control, and oversight of the project for the yoga program on sustained attention and discrimination function in ADHD children. The program for quality assurance plan in this study is used to control and monitor the quality aspects of the project activities. The Quality Assurance Project Plan is described including roles, responsibilities, and methodologies for ensuring compliance with the Research Ethic Committee of the National Taiwan University. The project for the yoga program on this research project is follow the requirements set forth in the Quality Assurance Plan as following:

***Program Title:*** Effects of an 8-Week Yoga Program on Sustained Attention and Discrimination Function in Children with Attention Deficit Hyperactivity Disorder

***Lead Organization:*** Graduate Institute of Sport Pedagogy, University of Taipei, No.101, Sec. 2, Zhongcheng Rd., Shilin Dist., Taipei City, Taiwan. Chung-Ju Huang, Project Manager/Coordinator, +886-2-82718288 EXT 5901

***Primary Contact:*** Chien-Chih Chou, Graduate Institute of Sport Pedagogy, University of Taipei, No.101, Sec. 2, Zhongcheng Rd., Shilin Dist., Taipei City, Taiwan. +886-2-82718288 EXT 5902

***Effective Date:*** This Quality Assurance Project Plan (QAPP) is effective from May 2013 to May 2015 unless otherwise revised, approved and distributed accordingly at an earlier date.

***Quality Assurance Project Plan:*** Sustained Attention and Discrimination Function Study of Children with ADHD in Taipei. Graduate Institute of Sport Pedagogy. Prepared for this research project, 50 pages plus appendices and attachments.

***Project Organization:*** The lines of communication between the participating entities, project organization and responsibilities are outlined in Table 1.

Table 1 Project Organization

| Position | Name | Responsibilities |
| --- | --- | --- |
| Contract Manager | Tzy-Yin Chen | Approve reports and invoices for payment. |
| Project Manager | Chung-Ju Huang | Project management and oversight. |
| Lead Scientist | Chien-Chih Chou | Advisory Roll; Data reporting |
| Project Coordinator | Chung-Ju Huang | Generation of a QAPP, Project coordination; ensures all laboratory activities are completed within proper timeframes. |
| Program QA Officer | Tzu-Yu Lin | Approve Quality Assurance Project Plan and oversee Sustained Attention and Discrimination Function Study of Children with ADHD projects’ QA |
| Laboratory Director | Chien-Chih Chou  Chung-Ju Huang | Organizing, coordinating, planning and designing research projects and supervising laboratory staff; Data validation, management and reporting |
| Sample Collection Coordinator | Ching-Yi Wu  Heng-I Hsiao  Meng-Hauan Hsieh  additional staff | Sampling coordination, operations, and implementing field-sampling procedures. |
| Technicians | Hsin-Yu Tu  Chia-Yin Lee | Conduct children with ADHD sustained attention and discrimination function. Non-invasive test in this study. |
| Sample Custodian | Wen-Yi Wang | Sample storage. |

***Expected Outcomes of the Study***

It is anticipated that yoga could enhance ADHD children’s sustained attention and discrimination function, all of which are expected to mediate the hypothesized improvement in clinical symptoms in exercise activity. Thus, it is also anticipated that yoga will prove beneficial for cognitive executive function during physical exercise.

***Dissemination of Results and Publication Policy***

For sponsored research in this study, the University of Taipei establishes the importance of disseminating research findings as one of its most important policies through its policy Openness in Research. That the principle of openness in research – the principle of freedom of access by all interested persons to the underlying data, to the processes, and to the final results of research – is one of overriding importance.” The Principal Investigator Responsibilities at University of Taipei also address the principle of openness in research. We followed the requirement of University of Taipei that provisions for fair and research results be included in its sponsored research agreements and has a process that allows us to place our findings in the public domain.

***Duration of the Project***

Participants in the yoga exercise group underwent an eight-week yoga exercise program that consisted of two 40-min sessions per week as an after school program. The study was conducted during May 13, 2013 to May 30, 2015.

***Problems Anticipated***

For problems anticipated, the procedure during yoga activities or pre-/post-tests did not meet minimal risk criteria. Therefore, there were no problems anticipated about harm or discomfort anticipated for physical or psychological functioning during our yoga activities or pre-/post-tests does in the research.

The manipulation of yoga activity followed the American Physical Therapy Association guidelines for working with children. Each lesson for yoga activity lasted for 40 min. For the yoga activity, moderate exercise consists of activities that result in a noticeable increase in breathing and heart rate, but can be comfortably sustained for ~ 40 minutes. During moderate intensity yoga exercise, participants should be able to easily carry on a conversation.

***Informed Consent Forms***

The informed consent form in this study consists of two parts: the information sheet and the consent certificate. The informed consent form in this study was approved by the Research Ethics Committee, National Taiwan University.

***Budget***

The budget was approximate 29,000 US dollars, and was support by Taipei city Government, 2012-2014.

***Other support for the Project***

No

***Collaboration with other scientists or research institutions***

No

***Links to other projects***

No

***Curriculum Vitae of investigators***

Dr. Chou is a professor in the Graduate Institute of Sport Pedagogy at the University of Taipei. Dr. Chou received the Bachelor of Education degree in 1992 from the Taipei Physical Education College, a Master of Education degree in 1996 from the Central Michigan University, USA, and his Doctor of Philosophy degree from the Florida State University, USA in 2001. Since 1993, Dr. Chou has been an administrator in the Chinese Taipei University Sports Federation, a middle/high school physical education teacher. Before coming to the University of Taipei, Dr. Chou has been a faculty member at the Taipei Physical Education College (2001-2013). He is the coordinator of university supervisors for student interns seeking certification in the area of physical education and teaches in the undergraduate sport pedagogy and fundamental exercise and sport science programs. His teaching expertise lies in the areas of sport pedagogy with emphasis on elementary methods and curriculum. His research agenda focuses on cognitive process through the tactical game activity in physical education. He also teaches classes for and supervises master’s and doctoral students studying sport pedagogy.

Dr. Huang is a professor in the Graduate Institute of Sport Pedagogy at the University of Taipei. Dr. Huang received the Bachelor of Education degree in 1991 from the National Taiwan of Normal University, Taiwan, a Master of Education degree in 1994 from the National Taiwan of Normal University, Taiwan, and his Doctor of Philosophy degree from the National Taiwan of Normal University, Taiwan in 2004. Since 1992, Dr. Huang has been a middle/high school physical education teacher and basketball coach, college physical education instructor, and college professor and researcher. Before coming to the University of Taipei, Dr. Huang has been a faculty member at the Taipei Physical Education College (1997-2013). Dr. Huang currently teaches undergraduate and graduate courses in sport and exercise psychology, as well as sport and exercise science. His research interests are centered around inhibitory function for special population, and how exercise and physical activity can favorably influence these outcomes in people. With the belief that more physical activity is better than less, and that any physical activity is better than none, Dr. Huang plans to determine factors that make exercise training an effective treatment method for some individuals, but not for others.

***Other research activities of the investigators***

No

***Financing and Insurance***

The investigators, Chien-Chih Chou, is responsible for paying for research related costs during the period of this study, and responsible for paying for injuries in case of accident.
