# Supplementary material for: Effects of an 8-week yoga program on sustained attention and discrimination function in children with attention deficit hyperactivity disorder
Source: PeerJ. 2017 Jan 12;5:e2883. doi: 10.7717/peerj.2883 (PMC5237364; doi:10.7717/peerj.2883)
Supplement: Supplemental Information 3 [file peerj-05-2883-s003.docx]

| **Paper**  **Section/**  **Topic** | **Item No** | **Descriptor** | **Reported?** | |
| --- | --- | --- | --- | --- |
|  |  |  | √ | **Pg #** |
| **Title and Abstract** | | | | |
| Title and  Abstract | 1 | - Information on how unit were allocated to interventions | √ | Effects of an 8-Week Yoga Program on Sustained Attention and Discrimination Function in Children with Attention Deficit Hyperactivity Disorder. |
|  |  | - Structured abstract recommended |  |  |
|  |  | - Information on target population or study sample | √ | Children with Attention Deficit Hyperactivity Disorder between 8 and 12 years old |
| **Introduction** | | |  |  |
| **Background** | 2 | - Scientific background and explanation of rationale |  | Exercise science and cognitive executive function. |
|  |  | - Theories used in designing behavioral interventions | √ | The school-based ADHD children intervention was based on the theory of sustained attention and discrimination function. |
| **Methods** |  |  |  |  |
| **Participants** | 3 | - Eligibility criteria for participants, including criteria at different levels in recruitment/sampling plan (e.g., cities, clinics, subjects) |  | The inclusion criteria were as follows: children aged between 8 and 12 years old who had been diagnosed with ADHD by a psychiatric physician as well as by a school pediatrician according to the DSM-IV criteria and interviews with parents. The exclusion criteria were as follows: a) comorbid conditions such as conduct/oppositional defiant disorder, autism spectrum disorders, or serious affective disorders, b) a personal history of brain injury or neurological disorders, and c) currently taking sedatives or other mood altering medications other than the stimulants typically prescribed for ADHD. Moreover, all the various subtypes of ADHD (inattentive, hyperactivity/impulsivity, combined) were included so long as the participant met the other criteria, and regardless of whether he or she was receiving medication for it. |
|  |  | - Method of recruitment (e.g., referral, self-selection), including the sampling method if a systematic sampling plan was implemented | √ | Participants were recruited via flyers posted in relevant locations, referrals given to the children’s parents by their elementary schools, and a number of orientations conducted to introduce the project. |
|  |  | - Recruitment setting |  |  |
|  |  | - Settings and locations where the data were collected | √ | It was conducted in Taipei. |
| **Interventions** | 4 | - Details of the interventions intended for each study condition and how and when they were actually administered, specifically including: |  |  |
|  |  | - Content: what was given? |  | Yoga activity. |
|  |  | - Delivery method: how was the content given? | √ | Each lesson for yoga activity lasted for 40 min, twice a week for eight weeks. |
|  |  | - Unit of delivery: how were the subjects grouped during delivery? | √ | The intervention of yoga activity was delivered to small groups of 10-12 participants. |
|  |  | - Deliverer: who delivered the intervention? | √ | Each lesson for yoga activity was led by a nationally certified yoga instructor. |
|  |  | - Setting: where was the intervention delivered? | √ | The intervention of yoga activity was delivered in a dance studio with an average temperature of 24-26°C. |
|  |  | - Exposure quantity and duration: how many sessions or episodes or events were intended to be delivered? How long were they intended to last? | √ | Each lesson for yoga activity lasted for 40 min, twice a week for eight weeks. |
|  |  | - Time span: how long was it intended to take to deliver the intervention to each unit? | √ | Each intervention session was to be delivered (the yoga activity session consisted of a 10-minute stretching and warming-up period followed by a 20-minute yoga activity, which included concentration and balance, improved attention, and breath and body awareness. Finally, each session ended with a 10-minute cooling-down period including balancing, flexibility, and relaxation exercises). |
|  |  | - Activities to increase compliance or adherence (e.g., incentives) |  |  |
| **Objectives** | 5 | - Specific objectives and hypotheses | √ | The hypothesis was that yoga exercise could benefit the sustained attention and discrimination function of children with ADHD by using the Visual Pursuit Test and the Determination Test. |
| **Outcomes** | 6 | - Clearly defined primary and secondary outcome measures |  |  |
|  |  | - Methods used to collect data and any methods used to enhance the quality of measurements | √ | Computer version of Visual Pursuit Test and the Determination Test was self-administered instrument. |
|  |  | - Information on validated instruments such as psychometric and biometric properties |  |  |
| **Sample size** | 7 | - How sample size was determined and, when applicable, explanation of any interim analyses and stopping rules | √ | Forty-nine participants were recruited via flyers posted in relevant locations, referrals given to the children’s parents by their elementary schools, and a number of orientations 100  conducted to introduce the project. |
| **Assignment Method** | 8 | - Unit of assignment (the unit being assigned to study condition, e.g., individual, group, community) | √ | Assignment method: All the children were assigned to one of two groups according to their school districts: the yoga exercise group (*n* = 24) and the control group (*n* = 25). |
|  |  | - Method used to assign units to study conditions, including details of any restriction (e.g., blocking, stratification, minimization) |  |  |
|  |  | - Inclusion of aspects employed to help minimize potential bias induced due to non-randomization (e.g., matching) |  |  |
| **Blinding (masking)** | 9 | - Whether or not participants, those administering the interventions, and those assessing the outcomes were blinded to study condition assignment; if so, statement regarding how the blinding was accomplished and how it was assessed. | √ | The staff member performing the assessments was not involved in implementing any aspect of the intervention and knew the participants only by their study identifier number. |
| **Unit of analysis** | 10 | - Description of the smallest unit that is being analyzed to assess intervention effects (e.g., individual, group, or community) |  |  |
|  |  | - If the unit of analysis differs from the unit of assignment, the analytical method used to account for this (e.g., adjusting the standard error estimates by the design effect or using multilevel analysis) | √ | Since analyses were performed at the individual level and Children with ADHD were assigned to either yoga exercise or regular physical activity, a prior estimate of any potential confounds would be homogenous for both the exercise and control groups to compare between the two groups. |
| **Statistical Methods** | 11 | - Statistical methods used to compare study groups for primary methods outcome(s), including complex methods of correlated data | √ | The effects of yoga exercise on the performance in the Visual Pursuit Test and the Determination Test were examined by 2 (Group: exercise, control) × 2 (Time: pre-test, post-test) mixed design ANOVAs. Following the ANOVAs, multiple comparisons with Bonferroni-Holm adjustments were applied to control for experiment-associated inflation of type 1 error for small sample sizes. |
|  |  | - Statistical methods used for additional analyses, such as a subgroup analyses and adjusted analysis |  |  |
|  |  | - Methods for imputing missing data, if used |  |  |
|  |  | - Statistical software or programs used | √ | SPSS 18.0 was used. |
| **Results** | | | | |
| **Participant flow** | 12 | - Flow of participants through each stage of the study: enrollment, assignment, allocation, and intervention exposure, follow-up, analysis (a diagram is strongly recommended) | √ | See Figure 1 in the manuscript. |
|  |  | - Enrollment: the numbers of participants screened for eligibility, found to be eligible or not eligible, declined to be enrolled, and enrolled in the study | √ |  |
|  |  | - Assignment: the numbers of participants assigned to a study condition | √ |  |
|  |  | - Allocation and intervention exposure: the number of participants assigned to each study condition and the number of participants who received each intervention | √ |  |
|  |  | - Follow-up: the number of participants who completed the follow-up or did not complete the follow-up (i.e., lost to follow-up), by study condition | √ |  |
|  |  | - Analysis: the number of participants included in or excluded from the main analysis, by study condition | √ |  |
|  |  | - Description of protocol deviations from study as planned, along with reasons |  |  |
| **Recruitment** | 13 | - Dates defining the periods of recruitment and follow-up | √ | The periods of recruitment in this study was conducted during May 13, 2013 to June 30, and the study was conducted during May 13, 2013 to May 30, 2015. |
| **Baseline Data** | 14 | - Baseline demographic and clinical characteristics of participants in each study condition | √ | No significant was found differences between the groups in terms of weight, height, body mass index, age, intelligence quotient (IQ), and physical fitness. In addition, in terms of gender, grade, type, and medicine intake for both groups, suggesting that the two groups were homogenous. |
|  |  | - Baseline characteristics for each study condition relevant to specific disease prevention research | √ | Visual pursuit and determination was used for testing accuracy rate and reaction time. |
|  |  | - Baseline comparisons of those lost to follow-up and those retained, overall and by study condition |  |  |
|  |  | - Comparison between study population at baseline and target population of interest |  |  |
| **Baseline equivalence** | 15 | - Data on study group equivalence at baseline and statistical methods used to control for baseline differences | √ | Baseline equivalence: the intervention and comparison groups did not statistically differ with respect to demographic data (weight, height, body mass index, age, IQ, physical fitness, gender, grade, type, and medicine intake; p > .05 for each group), but the yoga exercise group yielded a higher accuracy rate and reaction time at the post-test than the control group (p = .010), along with no group differences observed at the pre-test. Furthermore, the exercise group reported an increased accuracy rate and reaction time after the yoga intervention (p = 0.045), while no change in the accuracy rate was found for the control group (p = .397). |
| **Numbers analyzed** | 16 | - Number of participants (denominator) included in each analysis for each study condition, particularly when the denominators change for different outcomes; statement of the results in absolute numbers when feasible |  |  |
|  |  | - Indication of whether the analysis strategy was “intention to treat” or, if not, description of how non-compliers were treated in the analyses | √ | The primary analysis was intention to treat and included all subjects as assigned with available 8-week outcome data (24 assigned to the exercise and 25 assigned to the Control group) |
| **Outcomes and estimation** | 17 | - For each primary and secondary outcome, a summary of results for each estimation study condition, and the estimated effect size and a confidence interval to indicate the precision |  |  |
|  |  | - Inclusion of null and negative findings |  |  |
|  |  | - Inclusion of results from testing pre-specified causal pathways through which the intervention was intended to operate, if any |  |  |
| **Ancillary analyses** | 18 | - Summary of other analyses performed, including subgroup or restricted analyses, indicating which are pre-specified or exploratory |  |  |
| **Adverse events** | 19 | - Summary of all important adverse events or unintended effects in each study condition (including summary measures, effect size estimates, and confidence intervals) |  |  |
| **DUSCUSSION** | | | | |
| **Interpretation** | 20 | - Interpretation of the results, taking into account study hypotheses, sources of potential bias, imprecision of measures, multiplicative analyses, and other limitations or weaknesses of the study |  |  |
|  |  | - Discussion of results taking into account the mechanism by which the intervention was intended to work (causal pathways) or alternative mechanisms or explanations |  |  |
|  |  | - Discussion of the success of and barriers to implementing the intervention, fidelity of implementation |  |  |
|  |  | - Discussion of research, programmatic, or policy implications |  |  |
| **Generalizability** | 21 | - Generalizability (external validity) of the trial findings, taking into account the study population, the characteristics of the intervention, length of follow-up, incentives, compliance rates, specific sites/settings involved in the study, and other contextual issues |  |  |
| **Overall Evidence** | 22 | - General interpretation of the results in the context of current evidence and current theory | √ | Our positive findings are in accordance with previous studies demonstrating beneficial effects of a physical exercise program on attention and inhibition in children with ADHD. It is particularly significant that alternative therapies such as yoga exercise can be complementary to behavioral interventions for children with attention and inhibition problems. |
